# Progress in Research on the Effect of Melatonin on Postoperative Cognitive Dysfunction in Older Patients

**DOI:** 10.3389/fnagi.2022.782358

**Published:** 2022-03-09

**Authors:** Yuqing Wei, Chunlu Zhang, Danyang Wang, Chengping Wang, Lin Sun, Peng Chen

**Affiliations:** Department of Anesthesiology, China-Japan Union Hospital, Jilin University, Changchun, China

**Keywords:** melatonin, perioperative neurocognitive disorders(PND), postoperative cognitive dysfunction(POCD), older patients, circadian rhythms and sleep

## Abstract

Postoperative cognitive dysfunction (POCD) is a common complication of the central nervous system in elderly patients after operation. It will prolong the length of stay, reduce the independence and quality of daily life, and increase the risk of death. However, at present, there is a lack of safe and effective ideal drugs for the prevention and treatment of POCD. Melatonin is one of the hormones secreted by the pineal gland of the brain, which has the functions of regulating circadian rhythm, anti-inflammation, anti-oxidation, anti-apoptosis, and so on. Some recent studies have shown that MT can prevent and treat POCD by adjusting circadian rhythm, restoring cholinergic system function, neuroprotection, and so on. This article will introduce POCD, melatonin and the mechanism of melatonin on POCD, respectively, to provide a basis for clinical prevention and treatment of POCD in the elderly.

## Introduction

Postoperative cognitive dysfunction (POCD) is defined as a new cognitive disorder after anesthesia and operation, which is characterized by impaired personality, social ability, and cognitive ability after the operation and can last for days, months, or years. It is a common complication of the central nervous system in elderly patients after operation (Evered and Silbert, 2018). POCD is the result of various mechanisms, such as neuroinflammation, oxidative stress, dysfunction of neurotransmitters or their receptors, circadian rhythm disorders, iron overload (Gögenur et al., 2007; Li et al., 2016; Netto et al., 2018; Luo et al., 2019; Wang C. M. et al., 2021). At present, the drugs for the prevention and treatment of POCD include non-steroidal anti-inflammatory drugs (parecoxib, acetaminophen, ibuprofen), anesthesia-related drugs (dexmedetomidine, etomidate, oxycodone), ulinastatin, desferrioxamine, antipsychotics, and so on (Zhou et al., 2019; Gan et al., 2020; Huang et al., 2020; Yu and Xie, 2020; Zhao et al., 2020; Duan et al., 2021). However, there is still a lack of safe and effective ideal drugs. Melatonin (MT) is one of the hormones secreted by the brain’s pineal gland and has the effect of adjusting circadian rhythms, anti-inflammatory, antioxidant, anti-apoptosis, etc. (Opie and Lecour, 2016). Recent studies have shown that MT can play a particular role in preventing and treating POCD in the elderly. This paper aims to summarize these articles to provide a basis for clinical prevention and treatment of POCD in the elderly.

## Postoperative Cognitive Dysfunction

In 2018, POCD was renamed perioperative neurocognitive disorders (PND), including all perioperative cognitive changes. According to the onset time, PND was divided into five categories: preoperative cognitive dysfunction, postoperative delirium (POD), delayed neurocognitive recovery (DNR), postoperative neurocognitive disorders, and cognitive dysfunction after 12 months of surgery. Since previous basic and clinical studies all used the concept of POCD, this paper still used POCD to describe the effect of melatonin on postoperative cognitive function.

POCD is associated with a variety of preoperative, intraoperative, and postoperative factors, such as age, low level of education, preoperative cognitive dysfunction, sleep disorder, history of diabetes and hyperglycemia, vitamin D deficiency, prolonged operation time, elevated C-reactive protein after the operation (Borozdina et al., 2018). Due to the different experimental design and evaluation methods, the incidence of POCD is also different. It is reported that during minor surgery, the incidence of POCD within 7 days after operation was 6.8%, and the incidence of POCD within 3 months after operation was 6.6% (Canet et al., 2003). After major non-cardiac surgery, the incidence of POCD at discharge was 41.4%, compared with 12.7% at 3 months. Patients with POCD at discharge are more likely to die within 3 months after operation. Similarly, patients with persistent POCD for the first 3 months after surgery had a higher risk of death in the first year after surgery (Monk et al., 2008). POCD can prolong hospital stay, increase mortality, reduce the independence and quality of daily life, increase the risk of leaving the labor market prematurely and dependence on social transfer payments (Steinmetz et al., 2009; Deiner et al., 2020). Therefore, in clinical work, we need to pay attention to the evaluation of the perioperative cognitive status of elderly patients and take a variety of effective methods to prevent and treat POCD.

## Melatonin

Melatonin is a neuroregulatory hormone synthesized by pineal cells, which has the functions of regulating circadian rhythm, scavenging free radicals, antioxidation, anti-inflammation, and immune regulation. Studies have shown that people with neurological diseases have lower levels of melatonin. With its main characteristics, melatonin can prevent and treat many nervous system diseases such as Alzheimer’s disease, Parkinson’s disease, autism, multiple sclerosis, Huntington’s disease, amyotrophic lateral sclerosis, stroke, epilepsy (Chen et al., 2020; Gunata et al., 2020; Luo et al., 2020). POCD is a common central nervous system complication in elderly patients after operation, and its pathophysiological mechanism is similar to that of neurodegenerative diseases such as Alzheimer’s disease (Fodale et al., 2010; Duan et al., 2018). Therefore, it is found that the physiological characteristics of melatonin can also play an important role in the prevention and treatment of POCD (Song et al., 2018; Yang et al., 2020; Li et al., 2021).

## The Role of Melatonin in the Prevention and Treatment of Postoperative Cognitive Dysfunction in Elderly Patients

### Melatonin and Circadian Rhythm

Sleep and circadian rhythm disorder are common in elderly patients after major surgery (van Zuylen et al., 2021). Circadian rhythm coordinates almost all physiological functions and is related to the development of many diseases. In order to study the relationship between circadian rhythm disorder and POCD, the subjective and objective sleep quality and cognitive function of 36 patients undergoing major abdominal surgery were measured. It was found that POCD was related to poor subjective sleep quality and more arousal (Gögenur et al., 2007). Another study found that the incidence of POCD was significantly increased in patients with fluctuations in melatonin metabolite 6-hydroxy sulfate melatonin (6-SMT) after large-scale non-cardiac surgery, suggesting that the occurrence of POCD is related to fluctuations in endogenous melatonin levels (Wu et al., 2014). Melatonin is a kind of central circadian rhythm regulator, and the normal melatonin rhythm is involved in regulating the sleep-awakening cycle to some extent. All in all, there is ample evidence that melatonin production is disturbed after surgery, that perioperative sleep disorders increase the risk of POCD, and that synchronization disorders between sleep-awakening cycles and melatonin rhythms can lead to cognitive impairment (Wright et al., 2006; Song et al., 2021; Wang X. et al., 2021).

The basic research found that after isoflurane anesthesia, the clock gene expression phase and peak time in mice were advanced or delayed, and the circadian rhythm disorder and spatial memory ability decreased in mice. However, the mice were pretreated with 10 mg/kg melatonin for 7 consecutive days before anesthesia. The results showed that melatonin could restore the desynchronization of clock gene expression induced by isoflurane and improve aged mice’s circadian rhythm disorder and spatial memory impairment (Song et al., 2018). In addition, after sevoflurane anesthesia, the expression level of the clock gene in various organs of mice was also affected (Ohe et al., 2011; Mori et al., 2014). Jia et al. (2021) gave mice 10 mg/kg melatonin for 7 consecutive days before anesthesia and found that melatonin improved the circadian rhythm disorder caused by operation under sevoflurane anesthesia in elderly mice. The above studies show that anesthesia can cause circadian rhythm disorder and POCD by changing the expression level and rhythm of clock genes, while melatonin can restore circadian rhythm by promoting the resynchronization of clock gene expression rhythm and prevent and treat cognitive dysfunction caused by anesthesia. Imai et al. (2020) found that desflurane anesthesia changed the expression of the clock gene in the suprachiasmatic nucleus (SCN) and caused the phase shift of circadian rhythm, and these changes were related to the time of anesthesia day. Kadota et al. (2012) found that sevoflurane anesthesia inhibited the expression of mPer2 in SCN in a time-dependent manner, but no time-dependent effect on circadian rhythm was found. POCD and sleep disorders are related to circadian rhythm disorders. General anesthetics affect the development of these postoperative complications by changing the expression of clock genes in SCN. The above studies have found that the time of operation under inhalation anesthesia may affect the expression of clock genes, but whether circadian rhythm disorders and POCD are related to the time of anesthesia day need to be further studied. Other studies found that after long-term isoflurane anesthesia, spatial memory impairment and sleep-awakening rhythm disorder occurred in mice. At the same time, the expression of NR2B (N-methyl-D-aspartate receptor 2B subunit) and CREB (cAMP response element-binding protein) in the hippocampus decreased gradually. CREB is an important transcription factor in memory formation (Kida and Serita, 2014). For 7 days before anesthesia, melatonin pretreatment with 10 mg/kg improved the disturbed sleep-awakening cycle and isoflurane-induced cognitive impairment and reversed the down-regulation of CREB and NR2B expression. Experiments have shown that sleep-awakening rhythm is involved in isoflurane-induced cognitive impairment, and melatonin can positively affect circadian rhythm normalization and cognitive reversal through the NR2B-CREB signal pathway (Xia et al., 2016). Liu et al. (2013) found that prophylactic use of melatonin 1 or 10 mg/kg for 14 days before anesthesia could alleviate the decrease of plasma/hippocampal melatonin levels induced by isoflurane and cognitive impairment in aged rats. Further study found that administration of melatonin 10 mg/kg 30 min before anesthesia could have the same effect, and its protective effect depended on hippocampal MT2-CREB signaling pathway (Liu et al., 2017). In short, melatonin improves the cognitive performance of animal models, but the specific molecular mechanism of melatonin restoring circadian rhythm and reducing cognitive dysfunction remains to be further studied.

Many clinical studies have also shown that preoperative application of melatonin can restore normal circadian rhythm, improve sleep quality and reduce the incidence of POCD in elderly patients. In a randomized controlled study, 129 patients over 65 years old were randomly divided into two groups. The experimental group received 1 mg oral melatonin 1 day before operation and 1 h before bedtime for 5 consecutive days after the operation. The control group was given a placebo. The results noted that compared with the preoperative value or the melatonin group, the MMSE score of the control group decreased significantly after the operation. In contrast, the MMSE score of the melatonin group remained unchanged 7 days after the operation. In addition, compared with the melatonin group, the postoperative subjective sleep quality and overall health status of the control group were significantly impaired (Fan et al., 2017). In short, both animal models and clinical subjects treated with melatonin showed better cognitive performance. However, most clinical trials have limitations because the follow-up time is short, and only the MMSE score is used as diagnostic criteria for POCD. At present, there are few clinical trials, and the experimental design also has a high heterogeneity, so more perfect clinical trials are needed to prove that melatonin can prevent and treat POCD by restoring circadian rhythm (Table 1).

**TABLE 1:** Summary of experiments related to melatonin for the prevention and treatment of postoperative cognitive dysfunction.

Mechanism of action	Study	Anesthetic drugs	Dose and timing of melatonin administration	Results
Adjusting circadian rhythms	Song et al., 2018	Isoflurane	Gavage at 10 mg/kg daily for 7 consecutive days before anesthesia	Melatonin could restore the desynchronization of clock gene expression induced by isoflurane and improve aged mice’s circadian rhythm disorder and spatial memory impairment.
	Ohe et al., 2011; Mori et al., 2014	Sevoflurane		Sevoflurane anesthesia reversibly suppressed the expression of the clock gene, Period2 (Per2), in the mouse suprachiasmatic nucleus (SCN).
	Jia et al., 2021	Sevoflurane	Intraperitoneal injection of 10 mg/kg melatonin daily for 7 consecutive days before anesthesia	Melatonin could ameliorate the circadian rhythm disorder caused by operation under sevoflurane anesthesia in elderly mice.
	Imai et al., 2020	Desflurane		Desflurane anesthesia altered the relative mRNA expression of four major clock genes (Per2, Bmal, Clock, and Cry1) in the SCN and caused a phase shift in the circadian rhythm, which were dependent on the time of day of anesthesia.
	Kadota et al., 2012	Sevoflurane		Sevoflurane anesthesia inhibited the expression of mPer2 in SCN in a time-dependent manner, but no time-dependent effect on circadian rhythm was found.
	Xia et al., 2016	Isoflurane	Gavage at 10 mg/kg daily for 7 consecutive days before anesthesia	Sleep-awakening rhythm is involved in isoflurane-induced cognitive impairment, and melatonin can positively affect circadian rhythm normalization and cognitive reversal through the NR2B-CREB signal pathway.
	Liu et al., 2013	Isoflurane	Intraperitoneal melatonin 10 or 1 daily for 14 consecutive days before anesthesia	Prophylactic melatonin attenuated isoflurane-induced decreases in plasma/hippocampal melatonin levels and cognitive impairment in aged rats.
	Liu et al., 2017	Isoflurane	Intraperitoneal injection of melatonin 10 mg/kg 30 min before anesthesia	Preventive melatonin attenuated isoflurane-induced cognitive impairment and its effects were depended on the hippocampal MT2-CREB signaling pathway.
	Fan et al., 2017	Spinal anesthesia	1 mg oral melatonin daily 1 h before bedtime 1 day before surgery and for another 5 consecutive days postoperatively.	Exogenous melatonin increased perioperative sleep quality and improved early POCD.
Restoring cholinergic system function	Ni et al., 2013	Isoflurane	Intraperitoneal injection of 10 mg/kg melatonin daily for 7 consecutive days before anesthesia	Melatonin could inhibit the production of A β, increase the expression of ChAT and protect the function of the cholinergic system.
	Corrales et al., 2013		Oral melatonin 0.5 mg daily for 5 months	Melatonin alleviated cholinergic degeneration by increasing ChAT and improved the spatial learning and memory ability of mice.
	Chen et al., 2018		Intraperitoneal injection of melatonin 10 mg/kg daily for 2 and 4 weeks.	Melatonin treatment increased ChAT, CHT, VAChT and M1R immunoreactivity and their protein levels in the septum and hippocampus, restored cholinergic system function, and improved spatial learning and memory.
	Labban et al., 2021		Intraperitoneal injection of melatonin 80 mg/kg daily for 8 weeks	Melatonin increased AChE levels as well as BDNF/CREB1 protein expression levels in the prefrontal cortex of mice and improved recognition memory and passive avoidance performance
Protecting the nervous system	Zhong et al., 2018	Propofol		Propofol could induce extensive neuronal apoptosis by down-regulating the PKA-CREB-BDNF signaling pathway, thus leading to long-term spatial learning and memory disorders.
	Hosseini et al., 2019	Propofol	Intraperitoneal injection of melatonin 10 mg/kg every other day for 28 days	Melatonin could improve mitochondrial dysfunction and reduce apoptosis in the aged rat brain, thereby reversing aging-induced learning and memory deficits.
	Li et al., 2021	Propofol	Intraperitoneal injection of melatonin 10 mg/kg 20 min before anesthesia	Melatonin could alleviate mitochondrial dysfunction, apoptosis, inactivation of PKA/CREB/BDNF signaling, and synaptic dysfunction, thereby improving propofol-induced cognitive dysfunction.
	Yuan et al., 2019	Isoflurane	Intraperitoneal melatonin 10 mg/kg daily for 14 consecutive days before anesthesia	Melatonin could inhibit neuroinflammation by suppressing mTOR signaling in the hippocampus of aged mice, thereby reducing isoflurane-induced cognitive impairment.
	Yang et al., 2020		Intraperitoneal injection of melatonin 75 or 150 mg/kg	Melatonin could ameliorate intestinal I/R-induced neuroinflammation and cognitive dysfunction by inhibiting TLR4/MyD88 signaling pathway in microglia.
	Palmer et al., 2020		Oral melatonin 20 mg daily about 1 h before bedtime for 10 days	Melatonin could improve the adverse effects of ACBC on cognitive function, sleep quality, and depressive symptoms through its neuroprotective effect.

### Melatonin and Cholinergic System

At present, studies have discovered that cognitive dysfunction is related to the dysfunction of neurotransmitters or receptors. The decrease of cholinergic system function and the regulation disorder of other neurotransmitters such as dopamine, norepinephrine, 5-hydroxytryptamine, glutamic acid, and γ-aminobutyric acid will lead to POCD (Zhang et al., 2020). The central cholinergic system regulates the brain’s advanced cognitive functions, such as memory, learning, dendritic branching, neuronal development, and differentiation. However, the ideal effect of anesthesia depends on the reduction of acetylcholine release and the inhibition of cholinergic transmission. Most drugs used during anesthesia combine nicotinic and toxic receptors and have profound effects on brain function through a series of synaptic and postsynaptic events (Fodale et al., 2006). With the increase of age, the number of cholinergic neurons in the brain, especially in the temporal lobe cortex and hippocampus, decreased, resulting in a gradual decline in various functions related to memory and learning. While anesthesia and surgical factors significantly reduced cholinergic neurons, aggravating the cognitive impairment caused by the above degeneration (Xu et al., 2019). In order to explore the effect of melatonin on neurodegenerative lesions induced by anesthesia, Ni et al. found that isoflurane anesthesia decreased the expression of choline acetyltransferase (ChAT) in the hippocampus, increased the production of A β, and led to cholinergic dysfunction. However, daily administration of 10 mg/kg melatonin in mice for 7 days before anesthesia could inhibit the production of A β, increase the expression of ChAT, protect the function of the cholinergic system and reduce neurodegenerative diseases (Ni et al., 2013). Corrales et al. (2013) gave 0.5 mg melatonin to Down syndrome mice every day for 5 months. The study found that melatonin alleviated cholinergic degeneration by increasing ChAT and improved the spatial learning and memory ability of mice (Corrales et al., 2013). Labban et al. (2021) gave 80 mg/kg melatonin daily to Alzheimer’s mice for 8 weeks. The results revealed that melatonin increased acetylcholinesterase (AChE) levels as well as BDNF/CREB1 protein expression levels in the prefrontal cortex of mice and improved recognition memory and passive avoidance performance. Chen et al. (2018) found that intraperitoneal injection of scopolamine in mice significantly reduced the protein levels and immunoreactivity of ChAT, high-affinity choline transporter (CHT), vesicle acetylcholine transporter (VAChT), and M1 receptor (M1R) in the septum and hippocampus, and impaired spatial learning and memory. Treatment with melatonin increased the immunoreactivity and protein levels of ChAT, CHT, VAChT, and M1R in septal and hippocampal areas and improved spatial learning and memory (Chen et al., 2018). These studies demonstrated that melatonin improved cognitive dysfunction in various disease models by restoring the function of the cholinergic system. However, in the clinical context, whether melatonin can effectively prevent POCD through this mechanism remains further studied (Table 1).

### Melatonin and Neuroprotection

Neuroinflammation is considered to be the essential pathogenesis of POCD. It destroys the permeability of the blood-brain barrier and promotes the activation of microglia. Microglia produce many inflammatory factors such as free radicals, reactive oxygen species (ROS), reactive nitrogen (RNS), leading to oxidative stress. Oxidative stress induces mitochondrial dysfunction by damaging lipid, protein, and DNA in the hippocampus (HIP) and prefrontal cortex (PFC), reduces ATP production, and finally leads to neural cell damage. Neuroinflammation and oxidative stress can also reduce the level of brain-derived neurotrophic factor (BDNF), while BDNF plays a vital role in promoting the synthesis and consolidation of new memory, resulting in memory impairment and cognitive dysfunction (Netto et al., 2018). Zhong et al. (2018) showed that propofol could induce extensive neuronal apoptosis by down-regulating the PKA-CREB-BDNF signaling pathway, thus leading to long-term spatial learning and memory disorders. Melatonin and its metabolites have a strong antioxidant capacity. They can resist oxidative stress and cell apoptosis by directly scavenging free radicals, indirectly stimulating antioxidant enzymes to inhibit oxidase activity, and reducing the formation of hydroxyl radicals through transition metal chelation related to Fenton/Haber-Weiss reaction (Reiter et al., 2016). Hosseini et al. (2019) and Li et al. (2021) have found that melatonin can reduce mitochondrial dysfunction by reducing the production of mitochondrial ROS, increasing mitochondrial membrane potential (MMP) and the level of mitochondrial ATP, up-regulate the expression levels of PKA, CREB, and BDNF to reverse PKA/CREB/BDNF signal inactivation, reduce neuronal apoptosis, and up-regulate the expression of synaptic proteins such as Synaxin and PSD95 to reduce synaptic dysfunction, thus improving cognitive dysfunction caused by propofol. Moreover, the mammalian target of rapamycin (mTOR) plays a vital role in maintaining brain function. Studies have shown that mTOR promotes learning and memory formation through synaptic enhancement dependent on protein synthesis, and the imbalance of mTOR can impair learning, memory, and social behavior in mice (Hoeffer and Klann, 2010). Yuan et al. (2019) found that 14 days before anesthesia, mice were given 10 mg/kg exogenous melatonin, which could reverse the decrease of melatonin levels in plasma and hippocampus after isoflurane exposure, inhibit the overexpression of p-mTOR, reduce the concentration of inflammatory factors such as TNF- α, IL-1 β, and IL-6, and improve memory and spatial learning impairment caused by isoflurane. Experiments have revealed that melatonin can inhibit neuroinflammation by inhibiting mTOR signal transduction in the hippocampus of elderly mice, thus alleviating isoflurane-induced cognitive impairment. Recently, many studies have shown a microbial-brain-gut axis connection between the brain and the intestinal tract, and the imbalance of intestinal flora will increase the risk of neurodegenerative diseases such as POCD (Jiang et al., 2019; Lin et al., 2020). Yang et al. (2020) administered 75 or 150 mg/kg melatonin to intestinal ischemia/reperfusion (I/R) rats. They found that melatonin can alleviate intestinal injury and pathological brain injury in intestinal I/R rats, reduce the levels of pro-inflammatory factors and oxidative stress in intestinal, plasma, and brain tissues, reduce apoptosis, inhibit the expression, and immunoreactivity of TLR4 and MyD88 in microglia of brain tissues, and improve the cognitive function of intestinal I/R rats. These results suggest that Melatonin can ameliorate intestinal I/R-induced neuroinflammation and cognitive dysfunction by inhibiting TLR4/MyD88 signaling pathway in microglia (Yang et al., 2020). In a randomized controlled study, 36 women who received adjuvant chemotherapy for breast cancer (ACBC) were randomly given 20 mg melatonin or placebo daily before and during the first cycle of ACBC. The results found that melatonin can improve the adverse effects of ACBC on cognitive function, sleep quality, and depressive symptoms through its neuroprotective effect on neuroplastic processes (Palmer et al., 2020). Anyway, in experimental animal models, melatonin has been proved to be very effective in neuroprotection and improving cognitive processes. However, the dose of melatonin involved in the experiment vary, and high doses of melatonin are also effective at low doses. Therefore, well-designed clinical studies are needed to explore the neuroprotective effect of melatonin and its dose selection (Table 1).

## Summary and Prospect

To sum up, melatonin shows advantages in preventing and treating POCD in elderly patients. Melatonin can prevent and treat POCD by adjusting circadian rhythm, restoring cholinergic system function, neuroprotection. However, at present, there are few basic and clinical trials of melatonin in the prevention and treatment of POCD. And there is a large heterogeneity between the experiments. The doses of melatonin involved in the experiments vary, and the fact that high doses of melatonin are equally effective at low doses is an important feature. Moreover, the pathogenesis and pathophysiological basis of POCD have not yet been clarified, and after the renaming, the evaluation method has changed. Therefore, we need further molecular biology research and clinical trials to explore the signal mechanism, therapeutic effect, optimal administration time and dose of melatonin in the treatment of POCD. Furthermore, we can provide new research clues for the research of perioperative neurocognitive disorders.

## Author Contributions

YW conceived the idea of the review and wrote the first draft. CZ, DW, CW, and LS collected the literature and wrote the different sections. PC provided technical guidance and editing support. All authors participated in the revision of the manuscript and approved the final version.

## Conflict of Interest

The authors declare that the research was conducted in the absence of any commercial or financial relationships that could be construed as a potential conflict of interest.

## Publisher’s Note

All claims expressed in this article are solely those of the authors and do not necessarily represent those of their affiliated organizations, or those of the publisher, the editors and the reviewers. Any product that may be evaluated in this article, or claim that may be made by its manufacturer, is not guaranteed or endorsed by the publisher.
